# Supplementary material for: Spatio-temporal clustering of Mountain-type Zoonotic Visceral Leishmaniasis in China between 2015 and 2019
Source: PLoS Negl Trop Dis. 2021 Mar 22;15(3):e0009152. doi: 10.1371/journal.pntd.0009152 (PMC8016304; doi:10.1371/journal.pntd.0009152)
Supplement: Attachment — Submitted filename: point to point-202012Li.docx [file pntd.0009152.s001.docx]

**Point-by-point responses**

<b>Key Review Criteria Required for Acceptance?</b></br></br>
As you describe the new analyses required for acceptance, please consider the following:

<b>Methods

-Are the objectives of the study clearly articulated with a clear testable hypothesis stated?

**Response:** Yes, we set up a hypothesis that there are several clusters in the selected areas.

-Is the study design appropriate to address the stated objectives?

**Response:** Yes, according to the objective of this manuscript, spatial epidemiology methods were used in this paper, including Joinpoint regression analysis, Spatial autocorrelation and Spatio-temporal clustering analysis and so on. These methods are appropriate for this study.

-Is the population clearly described and appropriate for the hypothesis being tested?

**Response:** Yes, the population data is clearly and meet the objective hypothesis.

-Is the sample size sufficient to ensure adequate power to address the hypothesis being tested?

**Response:** Yes, we think so because all of reported cases were collected in this study, the clear data was included in the analysis.

-Were correct statistical analysis used to support conclusions?

**Response:** Yes, the epidemiological data was analyzed using Joinpoint regression methods.

-Are there concerns about ethical or regulatory requirements being met?

**Response:** No, there are not ethical or regulatory requirements.

Reviewer #1: The statistical and models are well written but some conceptual information needs to be reviewed. For instance, it is not clear which variables were selected as input for the clustering model and why. Also, a general description of the study area is missing. Understanding the region and main attributes of the size provinces are essential for the discussion. Moreover, the mentioned data source for the samples is not included as a reference.

**Response:** thanks reviewer`s the concern. We check the information carefully, for the clustering model and variables, we selected the spatial autocorrelation and spatio-temporal clustering methods, which we used in former studies, they show a good performance on the diseases clustering analysis. And also, they usually are used in this type of studies. For the study area, we revised and wrote the region attributes of the provinces into the second paragraph of the manuscript now. and provide the reference of the information system.

Reviewer #2: (No Response)

Reviewer #3: The objectives for this study are clearly articulated, and I feel that the overall study design is appropriate for the stated objectives.

The population is clearly described and appropriate for this study.

The sample size is sufficient to support the conclusions drawn.

One of the questions that I have regarding the statistical analysis used is that three different types of cluster analysis were used to describe the clustering of this disease. What is the benefit of using the different types of cluster analysis? This research did not discuss how the different methods could contrast or support each other. Also some of the terms used is the results, specifically a grade 1 cluster, were not explained before hand.

**Response:** thanks reviewer`s the understanding for the objective, population and sample size. For the three different types of cluster analysis, we hope to use different clustering methods to find out more identified clusters, and the results show us that the clusters are consistent. The methods are useful for this type of studies. In the part of discussion, we added the explanation of effect using different methods.

<b>Results</b></br></br>
-Does the analysis presented match the analysis plan?

**Response:** Yes, we think so. According to the analysis plan, the trends analysis was conducted using Joinpoint model and the hotspot region was found out. The clustering analysis was conducted using different methods of spatial epidemiological methods, and also the results show us that the clusters are consistent.

-Are the results clearly and completely presented?

**Response:** Yes, the results are very clearly and completely presented in this manuscript.

-Are the figures (Tables, Images) of sufficient quality for clarity?

**Response:** Yes, the quality of figures (Tables, Images) are sufficient for publication.

Reviewer #1: In general, the results are disorganized. The annual incidences presented in the text do not match the total number (529) nor the labels in the graph in the first figure. It is noticeable, that whenever results are presented for the six provinces respectively, it creates confusion for the reader due to miswriting. For instance, 5 different values are written as hotspots of infection respectively for the 6 provinces. Regarding results discussion, it gets confused with the introduction. The first three paragraphs are not analyzing the results but reporting general and conceptual facts as in the introduction. The discussion needs to be rewritten. In addition, it is important to represent cities mentioned in the text on the map, therefore readers who do not know the region would not get lost in the discussion.

**Response:** many thanks to the reviewer`s comments on this manuscript. According to the advices, we modified some parts of the results.

For the frame of the results, we organized them with an epidemiological analysis procedure including the trends analysis and test using Joinpoint model; then the spatial-temperal clustering analysis using hotspot analysis, Satscan soft and Felxscan soft were used to find out more clusters. Therefore, the logical order of result is better to understand.

For the annual incidences, there is a miswriting of the annual reported case number of 2019, the right number is 122 and updated now. The total of reported case number is 529. And the labels in the figure 1 are right. Therefore, the number of annual reported cases are 82, 95, 113, 117 and 122, respectively.

For the discussion part, the introduction of the concept is deleted now and the parts of influence factors are retained because they are important for the disease distribution when we discussed later.

For the map, we adopted the routine method to show disease distribution region.

Reviewer #2: (No Response)

Reviewer #3: The analysis presented does match the analysis described in the methods.
The results are clearly presented, however, as mentioned in my comments for the methods, it was not made clear my three different cluster analysis methods were used when the results indicate very similar outcomes.  More information either in this section or the discussion about the comparison and contrasts between the methods would be beneficial, or reducing the number of cluster analysis methods used.
Overall, the figures present the information presented, however there are some small additions that I feel would help deliver the information better. First, I would include a map of study area within the context of China as a whole for audience members that may not be familiar with the locations of Chinese provinces.  Second, since the disease is a "Mountain-type" disease and the paper mentions that there are geographic and ecological variations in may be useful to include information on the elevation in the selected study area to help people unfamiliar with the provinces understand if the incidence areas are close to mountains or not. Third, some minor clarifications and improvements to the maps presented would present a clearer picture of the results. I think that the figures in general should include a neat line around each individual map, that the word "legend" should be removed from the legends, and the scale bars should be standardized to either 500 km or 1000 km.
Fourth, in Figure 5, a-e, it is not readily apparent what the circles on the map are indicating.  Is this a part of the SatScan analysis used?  In the main body of the text there is no indication of what these circles mean.

**Response:** many thanks to the reviewer`s comments and advices for this part. According to the advices, we modified some parts of the results as follows.

For the clustering methods, we adopted three spatial-temperal clustering methods to identify the clustering region. We hoped to find out more or consistent clustering regions, and the result show us they are effective.

We checked and re-draw the maps according to the advices now. The maps have been replaced with the new maps in the context.

First, the whole country map and study area were added in the figures which help reader to understand the location of the study areas.

Second, the maps of the clustering regions were redraw in a three-dimensional map so that the geographic and ecological variations can be presented in a better mode and to be well understood as a "Mountain-type" disease. This a very useful advice for us.

Third, for the legends, we optimized the map according to this advice.

Fourth, For the Fig 5, the circles are the clustering results of Satscan analysis, they are meaning different clustering regions in the different years.

<b>Conclusions</b></br></br>
-Are the conclusions supported by the data presented?</br>

**Response:** Yes, these data showed that the incidence of MT-ZVL was on the rise, especially in Shanxi Province, and the cluster areas are gradually moving to the junction of Shaanxi and Shanxi and the eastern part of Shanxi. The conclusion of resurgence of MT-ZVL transmission is supported by the data.

-Are the limitations of analysis clearly described?</br>

**Response:** Yes, the limitations of analysis clearly described now.

-Do the authors discuss how these data can be helpful to advance our understanding of the topic under study?</br>

**Response:** Yes, we have discussed these data can be helpful to understanding of the topic under study.

-Is public health relevance addressed?</br></br>

**Response:** Yes, resurgence of MT-ZVL transmission needs to be concerned and to be resolved.

Reviewer #1: The conclusion does not make a clear statement of limitations, recommendations, or relevance of the study. Neither supports the article's objective. It is included in the objective the will to indicate important insights identified through the study for developing interventions aimed at the disease in question. However, nothing is mention in the conclusion.

**Response:** many thanks to the reviewer`s comments on this manuscript. According to the advices, we rewrote the conclusions.

For limitations of the study, we added that the risk of kala-azar may be underestimated in this study as cases in remote rural areas may not have been reported to local CDC due to mild symptoms or traffic restrictions.

For recommendations, we suggest that attention should be paid to resurgence of the epidemic in areas, and vector biological investigation, transmission risk assessment and targeted control measures should be carried out in a timely manner.

Reviewer #2: (No Response)

Reviewer #3: There is material in the conclusion section in the general discussion of MT-ZVL that I feel would be better supported in the introduction of the paper rather than in the conclusions.  Specifically, I feel that the second to fifth paragraphs, starting with "Currently, there are three types of VL in China..." and continuing for the next two paragraphs, feels like it should be included in the introduction as a general description of the disease.  Aside from this, I feel that the conclusions are supported by the data and analysis presented, although a discussion of what differences are coming from the different cluster analyses used would be helpful.  
The limitations are clearly described.
The authors do discuss the relevance of the analysis to the current medical field and addresses the relevance to public health.

**Response:** many thanks to the reviewer`s comments and advices for this part. According to your comments, we have reorganized the discussion section to focus on the problems found in this study. The introduction of the concept is deleted now and the parts of influence factors are retained because they are important for the disease distribution when we discussed later.

<b>Editorial and Data Presentation Modifications?</b></br><br/>
Use  this section for editorial suggestions as well as relatively minor modifications of existing data that would enhance clarity. If the only modifications needed are minor and/or editorial, you may wish to recommend “Minor Revision” or “Accept”. 

Reviewer #1: The map panels must be reviewed. It is not clear, neither on the image nor in the figure's description, the referenced year for each map. For maps with the same legend (eg. Fig 4), I suggest adding a bigger legend only once. Colors' differences between groups in Figures 5 and 6 are not clear - different colors would be better for highlighting groups than a gradient of a unique color. It is important to standardize the clusters' names in text and figures. It is sometimes referred to as level or sometimes as a grid. Moreover, it is necessary to review the references. In general, it does not follow the right pattern for referencing digital media and websites.

**Response:** many thanks to the reviewer`s comments and advices for this part. We redraw the maps as we can. Because these figures are drawn one by one, the legend was included in every small map. For fig 5 and 6, they show us the spatial clustering areas in different years using satscan and flexscan soft. They are all the spatial clustering results so that we adopt same color. We checked the references.

Reviewer #2: (No Response)

Reviewer #3: As discussed in the results section, there are some general points about the maps that will help with the data presentation:
Overall, the figures present the information presented, however there are some small additions that I feel would help deliver the information better.  First, I would include a map of study area within the context of China as a whole for audience members that may not be familiar with the locations of Chinese provinces.  Second, since the disease is a "Mountain-type" disease and the paper mentions that there are geographic and ecological variations in may be useful to include information on the elevation in the selected study area to help people unfamiliar with the provinces understand if the incidence areas are close to mountains or not. Third, some minor clarifications and improvements to the maps presented would present a clearer picture of the results.  I think that the figures in general should include a neat line around each individual map, that the word "legend" should be removed from the legends, and the scale bars should be standardized to either 500 km or 1000 km.
Fourth, in Figure 5, a-e, it is not readily apparent what the circles on the map are indicating.  Is this a part of the SatScan analysis used?  In the main body of the text there is no indication of what these circles mean.

**Response:** many thanks to the reviewer`s comments and advices. We checked and re-draw the maps according to the advices now. The maps have been replaced with the new maps in the context.

First, the whole country map and study area were added in the figures which help reader to understand the location of the study areas.

Second, the maps of the clustering regions were re-draw in a three-dimensional map so that the geographic and ecological variations can be presented in a better mode and to be well understood as a "Mountain-type" disease. This a very useful advice for us.

Third, for the legends, we optimized the map according to this advice.

Fourth, For the Fig 5, the circles are the clustering results of Satscan analysis, they are meaning different clustering regions in the different years.

<b>Summary and General Comments</b></br></br>
Use this section to provide overall comments, discuss strengths/weaknesses of the study, novelty, significance, general execution and scholarship. You may also include additional comments for the author, including concerns about dual publication, research ethics, or publication ethics. If requesting major revision, please articulate the new experiments that are needed.

Reviewer #1: The article lacks a discussion on how its results impact society and local institutions of disease control.

**Response:** many thanks to the reviewer`s comments and advices for this part. At the end of the paper, we propose to further strengthen the prevention and treatment of visceral leishmaniasis in key areas, pay attention to the resurgence of the epidemic, carry out timely vector investigation, spread risk assessment.

Reviewer #2: This is interesting research to explore the dynamics of Mountain-type Zoonotic Visceral Leishmaniasis in China between 2015 and 2019. The authors did a great work to analyze the data by using some modern analysis methods. The manuscript is well written and acceptable. However, I have some comments for the authors.

1.In material and methods section, how long was the time interval in your data acquisition? Monthly or Yearly? If the MT-ZVL incidence is annual data, how to ensure validity in the Joinpoint model by limited values? 
T-test is not suitable because the data within a certain period of time was dependent, unless chi square test checked its independence. Or use chi square test to check incidence of the same year between 6 provinces.
2.In spatial autocorrelation analysis, which disease indicator was employed to calculate c? It should be clarified by illustrative statements.
3.In results section, for hotspots, it should be explained more clearly in methods section about the map. And the year should be annotated in the legend.

**Response:** thanks to the reviewer`s comments. For the trends analysis, the annual data was observed in the Joinpoint model, although there were only five years data, the trends of the incidences of every provinces were analyzed respectively. Therefore, the data and T-test were used here.

The Moran's I index was used to determine whether there was a spatial correlation of VL. The results showed that the Moran's I values of the incidence of VLS in dogs from 2015 to 2019 were statistically significant. Then, local Getis-Ord Gi*, SaTScan and FlexScan were used to analyze the cluster areas of the disease.

The years have be added into legends of the maps.

Reviewer #3: I have no additional comments
